# Widespread myocardial dysfunction in COVID-19 patients detected by myocardial strain imaging using 2-D speckle-tracking echocardiography

**DOI:** 10.1038/s41401-020-00595-z

**Published:** 2021-01-28

**Authors:** Rui Li, Hong Wang, Fei Ma, Guang-lin Cui, Li-yuan Peng, Chen-ze Li, He-song Zeng, Ali J. Marian, Dao-wen Wang

**Affiliations:** 1 Division of Cardiology, Department of Internal Medicine and Hubei Key Laboratory of Genetics and Molecular Mechanism of Cardiologic Disorders, Tongji Hospital, Tongji Medical College, Huazhong University of Science and Technology, Wuhan, 430030 China; 2 The Center for Cardiovascular Genetics, The Brown Foundation Institute of Molecular Medicine, The University of Texas Health Science Center, Houston, TX 77030 USA

**Keywords:** COVID-19, global longitudinal strains, 2-D STE, SARS-CoV-2, myocardial injury, SpO_2_, hsCRP, IL-6

## Abstract

COVID-19 is a multiorgan systemic inflammatory disease caused by SARS-CoV-2 virus. Patients with COVID-19 often exhibit cardiac dysfunction and myocardial injury, but imaging evidence is lacking. In the study we detected and evaluated the severity of myocardial dysfunction in COVID-19 patient population using two-dimensional speckle-tracking echocardiography (2-D STE). A total of 218 consecutive patients with confirmed diagnosis of COVID-19 who had no underlying cardiovascular diseases were enrolled and underwent transthoracic echocardiography. This study cohort included 52 (23.8%) critically ill and 166 noncritically ill patients. Global longitudinal strains (GLSs) and layer-specific longitudinal strains (LSLSs) were obtained using 2-D STE. Changes in GLS were correlated with the clinical parameters. We showed that GLS was reduced (<−21.0%) in about 83% of the patients. GLS reduction was more common in critically sick patients (98% vs. 78.3%, *P* < 0.001), and the mean GLS was significantly lower in the critically sick patients than those noncritical (−13.7% ± 3.4% vs. −17.4% ± 3.2%, *P* < 0.001). The alteration of GLS was more prominent in the subepicardium than in the subendocardium (*P* < 0.001). GLS was correlated to mean serum pulse oxygen saturation (SpO_2_, RR = 0.42, *P* < 0.0001), high-sensitive C-reactive protein (hsCRP, *R* = −0.20, *P* = 0.006) and inflammatory cytokines, particularly IL-6 (*R* = −0.21, *P* = 0.003). In conclusions, our results demonstrate that myocardial dysfunction is common in COVID-19 patients, particularly those who are critically sick. Changes in indices of myocardial strain were associated with indices of inflammatory markers and hypoxia, suggesting partly secondary nature of myocardial dysfunction.

## Introduction

Novel coronavirus disease 2019 (COVID-19) emerged in Wuhan in early December and became a pandemic. Acute myocardial injury, detected by elevated serum levels of cardiac biomarkers, such as cardiac troponin I (cTnI) and N-terminal pro-B-type natriuretic peptide (NT-proBNP), is one of the complications observed in COVID-19 patients. Earlier studies reported elevated serum biomarkers in ~10% of all COVID-19 patients admitted to an intensive care unit (ICU) and in up to one-third of the COVID-19 patients who were in critical condition [1–3]. A recent study specifically analyzed the prevalence of myocardial injuries in patients with COVID-19 and found elevated serum cTnI in 14.6% of the entire cohort and 62.5% of critical patients [4]. These findings indicated that acute myocardial injury affected a significant portion of hospitalized COVID-19 patients and is a likely determinant of adverse clinical outcomes. However, imaging evidence of cardiac injury was lacking in these reports.

Two-dimensional speckle-tracking echocardiography (2-D STE) is a quantitative method to assess global and regional myocardial function with high sensitivity. It is especially valuable in detecting subclinical cardiac dysfunction [5, 6], such as asymptomatic patients with diabetes, young hypertensives, and patients with nonobstructive coronary artery disease [7–10]. Strain- and/or layer-specific strain analysis by 2-D STE could facilitate the diagnosis of acute myocarditis [11–14], especially in clinical situations where access to cardiac magnetic resonance (CMR) and/or endomyocardial biopsy (EMB) is limited.

In this study, we examined cardiac structure and function by echocardiography in a large cohort of hospitalized patients with COVID-19 in Wuhan, China. We examined global and layer-specific longitudinal strains (GLSs and LSLSs) by 2-D STE to quantify local myocardial function and compared the differences in myocardial strains between critical and noncritical COVID-19 cases to determine the clinical significance of cardiac dysfunction in the outcome of the disease. In addition, serum inflammatory cytokines and pulse oxygen saturation (SpO_2_) were measured, and associations with 2-D STE findings were analyzed to assess the potential contributions of systemic inflammation and hypoxemia to myocardial injury in COVID-19.

## Materials and methods

### Study design and population

This was a prospective cross-sectional study in a single center. Transthoracic echocardiographic (TTE) scans were performed randomly in 240 consecutive hospitalized patients with confirmed COVID-19 from March 15, 2020 to April 1, 2020 in Tongji Hospital, Wuhan, China. The study was approved by the Institutional Review Board of the Ethics Committee of Tongji Hospital. The requirement for informed consent was waived because the patient data were anonymous and COVID-19 was an emergency pandemic. The COVID-19 diagnosis was confirmed using high-throughput sequencing or a real-time reverse-transcriptase-polymerase-chain-reaction assay of nasal/pharyngeal swab specimens. Patients’ baseline data from clinical medical records were analyzed along with their TTE results. Patients who had a known history of or a TTE diagnosis of prior underlying cardiovascular diseases were excluded. Patients with poor echocardiographic imaging quality and arrhythmias that interfered with image processing were excluded as well. Of these patients, two had dilated cardiomyopathy, two had myocardial infarction, one had coronary artery disease with a history of percutaneous coronary intervention, two had valvular heart diseases, and fifteen had poor imaging quality or arrhythmias. The remaining 218 patients were enrolled in the final study. The control group consisted of 23 healthy volunteers who had no cardiopulmonary diseases according to their medical history, physical examination, electrocardiogram, chest X-ray, and echocardiography.

### Data collection

We obtained the clinical medical records, nursing records, laboratory findings, and radiological exams from the electronic medical charts for all enrolled patients from January 25, 2020, to April 1, 2020; the data cutoff date for the study was April 2, 2020. Data were recorded in the case record forms modified from the standardized International Severe Acute Respiratory and Emerging Infection Consortium case report form (https://isaric.tghn.org).

The demographics and baseline characteristics of all the enrolled COVID-19 patients included age, sex, hospitalization days, and chronic medical histories, including cardiovascular diseases, chronic pulmonary diseases, cerebrovascular diseases, hypertension, diabetes, chronic hepatic diseases, and malignancy. The hospitalization days were defined as days from admission to discharge or to the data cutoff date for the study. The laboratory variables consisted of a complete blood count, blood chemical analysis, liver and renal function, C-reactive protein (CRP), cTnI, NT-proBNP, and inflammatory markers (IL-6, IL-10, and TNF-α), which were all completed within 1 week from the day of the TTE scan. Chest computed tomography scans were reviewed by two independent physicians in respiratory medicine. Treatments were not discussed and thus not recorded in the study data. Medical treatments were recorded as well, including antiviral therapy, intravenous and oral use of corticosteroids, and use of dopamine or norepinephrine.

The severity of COVID-19 was determined according to the World Health Organization interim guidance [15] and the Clinical Guidance for COVID-19 Diagnosis and Treatment published by the National Health Commission of China [15]. Briefly, critical patients were defined as those who developed respiratory failure requiring mechanical ventilator support, patients who developed hypotension requiring vasopressors, and patients requiring ICU monitoring and treatments.

### Echocardiographic examinations and strain analysis by 2-D STE

TTE scans were typically performed toward the end of the hospitalization period. The TTE scan day was defined as the number of days from admission to the day when the TTE scan was performed. Vital signs, including blood pressure, heart rate and resting SpO_2_, and symptoms, including palpitation, dyspnea, and chest tightness, were recorded on the day when the TTE scan was performed.

For the TTE scan, a Vivid E95 ultrasound scanner (GE Vingmed; Horten, Norway) was used. Diastolic interventricular septum thickness (IVS) and left ventricular (LV) posterior wall thickness, LV end-diastolic dimensions (LVEDD), and end-systolic dimensions were measured from the parasternal long-axis view. LV ejection fraction (EF), LV end-diastolic volume (LVEDV), and end-systolic volume were calculated by the modified biplane Simpson method.

Two-D grayscale harmonic images and three consecutive cardiac cycles of each view were obtained, captured at a frame rate between 40 and 60 fps. LV strains were quantified using Echo Pac (version: 203.66; GE Vingmed; Horten, Norway). The methodology for longitudinal strain analysis was described previously in detail elsewhere [16]. Briefly, three apical views were used. Three endocardial points were applied in each apical view with two points at each side of the mitral annulus and a third point at the LV apex at the end-systolic period. The timing of aortic valve closure was determined automatically by the software and was visually confirmed and readjusted if necessary. Thereafter, the software automatically provided three lines along with endocardial, mid-myocardial, and epicardial layers, which followed each myocardial layer by a speckle-tracking algorithm. The tracking quality throughout the cardiac cycle was validated after adjusting the region of interest manually to include the entire myocardial layer if necessary. The peak systolic longitudinal strain values of each of the 17 LV segments defined by the American Heart Association LV model were then obtained by an automated algorithm. GLS was calculated as the mean of all 17 segmental strain values as described previously [17].

### Study outcomes

The outcomes of the study were death, length of hospitalization, and reduction in GLS and/or LV EF.

### Statistical analysis

Descriptive statistics were obtained for all study variables. Categorical variables are expressed as numbers (percentages), normally distributed continuous data as the mean with standard deviation and nonnormally distributed continuous data as the median with interquartile range (IQR). Continuous variables between groups were analyzed by the Student’s *t* test or Mann–Whitney U test according to their distribution. For paired comparisons, paired *t* tests or paired-sample Wilcoxon rank tests were performed for continuous variables, depending on the normality of the variables. Linear regression analysis was used to test for the possible association of GLS with cTnI, NT-proBNP, serum inflammatory markers and cytokines, SpO_2_, and medical treatment. Covariates such as age and sex were controlled in the multivariate regression model. All analyses were performed using SPSS version 19.0 software (SPSS, Inc., Chicago, IL, USA). Statistical tests were two-tailed, and a *P* value of <0.05 was considered statistically significant.

### Reproducibility

To investigate inter- and intrapersonal measurement reproducibility, measurements for all subjects were performed offline by two independent investigators. Intrapersonal agreement was measured 7 days later by the same investigator. The interclass correlation coefficients (ICCs) were calculated; the point estimates and the 95% confidence intervals (CIs) were reported.

## Results

### Clinical characteristics of the patients

Patients were enrolled in this study beginning on March 15, 2020, when most of the subjects were in their mid to late phases of hospitalization. The median hospital length was 28 (IQR: 16, 43) days, and the median time from admission to TTE scan was 24 (IQR: 14, 42) days (Tables 1 and 2). Of the 218 patients who met the inclusion criteria, 52 were critically ill (23.9%). The demographic and clinical characteristics of the patient cohort stratified by disease severity are described in Table 1. The mean age was 62 (IQR: 55, 69) years. The critically ill patients were significantly older than the noncritically ill patients (mean age: 61, IQR: 53, 68 vs. mean age: 64, IQR: 58, 73, *P* = 0.017). Males and females were equally represented in the total patient cohort; however, male patients comprised 68.5% of the critical patients as opposed to 47% of the noncritical patients (*P* = 0.046). The most common symptom was shortness of breath (40.8%). Only a small percentage of patients had symptoms consistent with suspicious cardiac conditions, including chest tightness (28.9%) and palpitation (2.8%). Heart rate was marginally faster in the critical patients than in the noncritical patients (95 ± 13 vs. 89 ± 16 beats/min, *P* = 0.011), and the mean systolic blood pressure was modestly higher in the critical patients. Resting SpO_2_ was markedly lower in the critical patients (81% ± 12% vs. 93% ± 4%, *P* < 0.001). There were no other differences in the clinical characteristics between the two groups. In terms of medical treatment, there was no difference in antiviral therapy between the two groups. However, the use of corticosteroids was higher in the critical group than in the noncritical group (intravenous use, 80.8% vs. 28.3%, *P* < 0.001; oral use, 32.7% vs. 18.1%, *P* < 0.05, respectively). The application of dopamine or norepinephrine was also higher in critical than in noncritical patients (19.2% vs. 0%, *P* < 0.001).

**Table 1:** Demographic, clinical characteristics, and outcomes of patients with COVID-19.

	Total (*n* = 218)	Noncritical (*n* = 166)	Critical (*n* = 52)	*P* value^a^
Demographic				
Age (years)	62 (54.8, 69.3)	61 (53, 68)	64 (58, 73)	0.017
Male, *n* (%)	118 (49.6)	78 (47)	33 (68.5)	0.046
Clinical presentation				
Palpitation, *n* (%)	6 (2.8)	6 (3.6)	0 (0)	0.340
Chest tightness, *n* (%)	63 (28.9)	50 (30.1)	13 (25.0)	0.477
Shortness of breath, *n* (%)	89 (40.8)	72 (43.4)	17 (32.7)	0.171
Signs				
SBP (mmHg)	135.0 ± 18.0	134.1 ± 17.8	137.7 ± 18.4	0.021
DBP (mmHg)	81.8 ± 11.5	81.2 ± 12.0	83.9 ± 9.3	0.103
HR (beat/min)	90 ± 15	89 ± 16	95 ± 13	0.011
SpO_2_ (%)	90 ± 9	93 ± 4	81 ± 12	<0.001
Blood and chemical tests				
WBC count (10^9^/L)	6.7 (5.1, 9.8)	6.2 (4.8, 8.1)	10.3 (6.6, 13.4)	<0.001
Neutrophil (%)	72.9 (60.1, 85.8)	66.9 (57.6, 79.9)	88.3 (80.3, 91.3)	<0.001
Lymphocyte (%)	15.9 (7.7, 27.0)	20.6 (11.0, 30.1)	6.9 (4.1, 10.4)	<0.001
Albumin (g/L)	35.0 ± 6.6	36.3 ± 6.6	31.3 ± 4.8	<0.001
Creatinine (mmol/L)	69.1 ± 24.2	66.5 ± 21.7	77.4 ± 29.7	0.017
Biomarkers for MI				
Peak cTnI (pg/mL)	4.5 (1.9, 15.7)	3.2 (1.9, 10.8)	15.6 (6.2, 58.4)	<0.001
>34.2 pg/mL, *n* (%)	23 (10.8)	8 (5.0)	15 (28.8)	<0.001
>34.2 and <100 pg/mL, *n* (%)	15 (6.8)	8 (5.0)	7 (13.5)	<0.001
Peak NT-proBNP (pg/mL)	173 (53, 563)	108 (36.5, 336.5)	582 (192.5, 1703)	0.004
>900 pg/mL, *n* (%)	32 (15.3)	14 (8.9)	18 (34.6)	<0.001
Creatine kinase (U/L)	54 (34, 89.5)	54 (36, 87)	55.5 (25, 145.8)	0.981
Inflammatory markers				
hsCRP (mg/L)	16.6 (2.6, 66.3)	6.4 (1.7, 45.6)	67 (41.2, 199.7)	<0.001
ESR (mm/h)	30 (14, 67)	19 (7.5, 55.5)	56 (30.5, 78)	<0.001
IL-6 (pg/mL)	6.1 (2.2, 23.9)	3.8 (1.6, 11.8)	32.7 (9.5, 109.9)	0.045
IL-10 (pg/mL)	5 (5, 7.7)	5 (5, 5.6)	7.1 (5, 14.3)	0.179
TNF-α (pg/mL)	8.5 (6.3, 11.3)	8.2 (6.2, 11)	10.2 (7.3, 16.2)	0.002
>50% involvement of chest CT, *n* (%)	137 (62.8)	89 (53.6)	48 (92.3)	<0.001
Medical treatment				
Antiviral therapy	184 (84.4)	138 (83.1)	46 (88.4)	0.358
Intravenous corticosteroid	89 (40.8)	47 (28.3)	42 (80.8)	<0.001
Oral corticosteroid	47 (21.6)	30 (18.1)	17 (32.7)	0.025
Dopamine/norepinephrine	10 (4.6)	0 (0)	10 (19.2)	<0.001
Outcomes				
Hospital length (days)	28 (16, 43)	27 (14, 43)	40 (28, 45)	<0.001
Death, *n* (%)	2 (0.9)	0 (0)	2 (3.4)	<0.001
^b^GLS < −21.0%, *n* (%)	181 (83.0)	130 (78.3)	51 (98)	<0.001
EF < 50%, *n* (%)	48 (22)	18 (10.8)	30 (57.6)	<0.001

Data are mean (SD) or median (IQR) for continuous variables or number (%) for categorized variables.

*SBP* systolic blood pressure, *DBP* diastolic blood pressure, *HR* heart rate, *GLS* global longitudinal strains, *EF* ejection fraction, *SpO*_*2*_ pulse oxygen saturation, *cTnI* cardiac troponin I, *hsCRP* high-sensitive C-reactive protein.

^a^*P* value calculated for critical vs. noncritical patients.

^b^−21.0% was the mean value of GLS for normal subjects (see Table 2).

**Table 2:** Echocardiography and myocardial longitudinal strain measurements in patients with COVID-19.

	Total (*n* = 218)	Noncritical (*n* = 166)	Critical (*n* = 52)	Normal (*n* = 23)	*P* value
^a^TTE day (days)	24 (14, 42)	23 (13, 42)	38 (27, 44)	–	<0.001^b^
Traditional parameters					
LVEDD (cm)	4.5 ± 0.4	4.5 ± 0.3	4.6 ± 0.5	4.5 ± 0.3	0.045^b^
LVEDV (mL)	76.4 ± 45.6	75.9 ± 49.8	78.0 ± 28.5	78.3 ± 13.5	0.939^b^
LVESD (cm)	3.1 ± 0.5	3.0 ± 0.4	3.3 ± 0.6	3.1 ± 0.5	<0.001^b^
LVESV (mL)	33.0 ± 15.1	30.4 ± 9.2	41.5 ± 24.5	28.9 ± 6.4	<0.001^b^
IVS	1.0 ± 0.2	0.9 ± 0.2	1.0 ± 0.2	0.8 ± 0.1	<0.001^b^
LVPW	1.0 ± 0.5	1.0 ± 0.5	1.0 ± 0.2	0.8 ± 0.1	0.326^b^
EF (%)	55.4 ± 9.2	57.8 ± 7.3	47.8 ± 10.4	62.9 ± 3.4	<0.001^b^
Pericardial effusion, *n* (%)	25 (11.5)	19 (11.4)	6 (11.5)	–	0.853^b^
GLS (%)	−16.5 ± 3.6	−17.4 ± 3.2	−13.7 ± 3.4	−21.0 ± 2.3	<0.001^b^
Layer-specific strains					
Endo (%)	−19.9 ± 4.1	−20.8 ± 3.5	−16.8 ± 4.6	−22.1 ± 2.3	<0.001^b^
Epi (%)	−15.0 ± 3.3	−15.8 ± 2.8	−12.6 ± 3.6	−18.0 ± 1.2	<0.001^b^
Epi/Endo	0.75 ± 0.04	0.76 ± 0.04	0.74 ± 0.06	0.82 ± 0.06	<0.001^c^
^d^GLS reduction	4.5 ± 3.6	3.6 ± 3.2	7.3 ± 3.4	–	<0.001^b^
^d^Endo reduction	2.4 ± 4.9	1.6 ± 4.6	4.9 ± 5.4	–	<0.001^b^
^d^Epi reduction	3.1 ± 3.5	2.3 ± 3.1	5.4 ± 3.6	–	<0.001^b^

Data are mean (SD) or median (IQR) for continuous variables or number (%) for categorized variables.

*IVS* interventricular septum thickness, *LV* left ventricle, *LVPW* LV posterior wall thickness, *LVEDD* LV end-diastolic dimensions, *LVESD* LV end-systolic dimensions, *LVEDV* LV end-diastolic volume, *LVESV* LV end-systolic volume, *GLS* global longitudinal strains, *Endo* subendocardium, *Epi* subepicardium, *EF* ejection fraction.

^a^TTE day was defined as days from admission to the day when echocardiography scan was performed.

^b^*P* value calculated for critical vs. noncritical patients.

^c^*P* value calculated for total patient vs. normal control.

^d^Mean strain values in normal subjects were used to calculate the reducing magnitude of GLS, subepicardial longitudinal strains, and subendocardial longitudinal strains.

### Laboratory test results

The myocardial injury biomarker hypersensitive cTnI was elevated in 23 patients (10.8%), including 15 critical cases (28.8%) and 8 noncritical cases (4.8%) (*P* < 0.001). The average level of cTnI was also higher in critical patients than in noncritical patients (15.6 (IQR: 6.2, 58.4) vs. 3.2 (IQR: 1.9, 10.8) pg/mL, *P* < 0.001). NT-proBNP was elevated in 32 patients (15.3%), including 18 critical (34.6%) vs. 14 noncritical patients (8.9%) (*P* < 0.001). In line with the elevation of cTnI, the average NT-proBNP level was also higher in the critical patients than in the noncritical patients (582, IQR: 192.5, 1703 vs. 108, IQR: 36.5, 336.5 pg/mL, respectively, *P* = 0.004).

Levels of inflammatory markers, including high-sensitivity CRP (hsCRP) and ESR, were much higher in the critical group than in the noncritical group (hsCRP: 67, IQR: 41.2, 199.7 vs. 6.4, IQR: 1.7, 45.6 mg/L, respectively, *P* < 0.001; ESR, 56, IQR: 30.5, 78 vs. 19, IQR: 7.5, 55.5 mm/h, respectively, *P* < 0.001). Inflammatory cytokines, including IL-6 and TNF-α, were also elevated in this COVID-19 cohort and were higher in critical patients than in noncritical patients (IL-6, mean: 32.7, IQR: 9.5, 109.9 vs. 3.8, IQR: 1.6, 11.8, pg/mL, respectively, *P* = 0.045; TNF-α, mean: 10.2, IQR: 7.3, 16.2 vs. mean 8.2, IQR: 6.2, 11 pg/mL, respectively, *P* = 0.002).

### Echocardiographic findings and longitudinal strain analysis

**Fig. 1:**
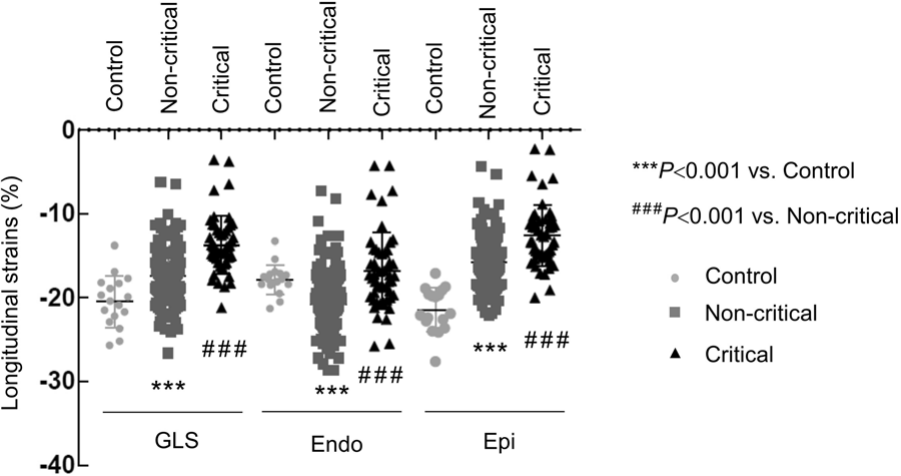
Comparison of GLSs, longitudinal strains in the subendocardium and longitudinal strains in the subepicardium in normal subjects, noncritical patients with COVID-19 and critical patients with COVID-19. GLSs global longitudinal strains, Endo subendocardium, Epi subepicardium. ***P value vs. control; ^###^*P* value vs. noncritical.

**Fig. 2:**
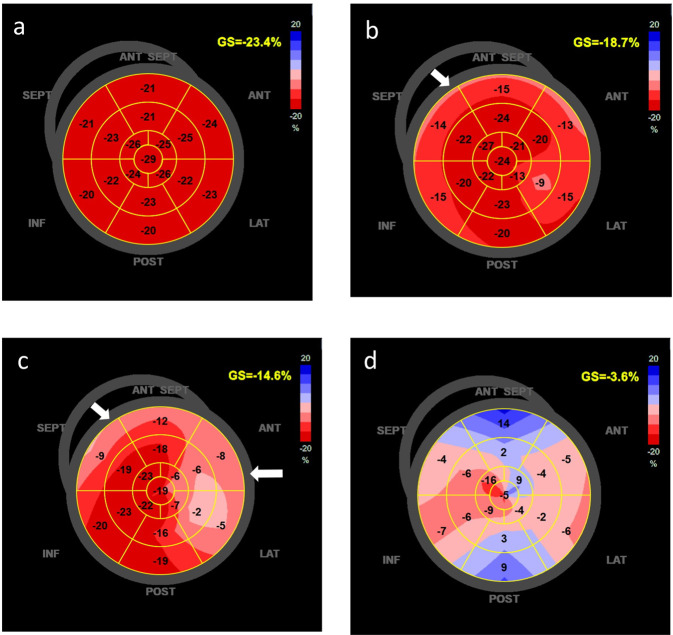
Representative images of GLS presented as “bull’s-eye”. (**a**) The GLS of a normal subject was −23.4%, (**b**) the GLS of a noncritical COVID-19 patient was −18.7%, (**c**) the GLS of a critical COVID-19 patient was −14.6%, (**d**) the GLS of a COVID-19 patient who died was −3.6%. The reduction in GLS was worse in the severe patient, and the alterations were more prominent in the basal-septal and basal-lateral regions of the LV (arrows).

Conventional echocardiographic parameters, GLS and LSLS indices in normal controls, noncritical patients, and critical patients are presented in Table 2. The longitudinal strains are shown in Fig. 1. Echocardiographic indices of LV size were normal in COVID-19 patients (LVEDD: 4.5 ± 0.4 cm and LVEDV of 76.4 ± 45.6 mL). The wall thickness was slightly thickened, particularly for the septum. The difference in the IVS was significant in the critical patients compared with the noncritical patients (1.0 ± 0.2 vs 0.9 ± 0.2 cm, *P* < 0.001). GLS and EF were reduced in both critical and noncritical cohorts in comparison with the normal control population. Overall, 83% of all patients had reduced GLS (<−21.0%), and 22% of all patients had reduced LV EF (<50%) (Table 1). The critically ill group had more patients with reduced GLS or reduced LV EF than the noncritical group (GLS: 98% vs. 78.3%, respectively, *P* < 0.001; EF: 57.6% vs. 10.8%, respectively, *P* < 0.001) (Table 1). Moreover, these indices were lower in the critical than in the noncritical patients (GLS: −13.7 % ± −3.4% vs. −17.4% ± −3.2%, respectively, *P* < 0.001; EF: 47.8% ± 10.4% vs. 57.8% ± 7.3%, respectively, *P* < 0.001). Representative bull’s eye images of GLS in a normal subject, a noncritical patient, a critical patient, and a patient for whom the TTE scan was performed 2 days before death are shown in Fig. 2.

**Fig. 3:**
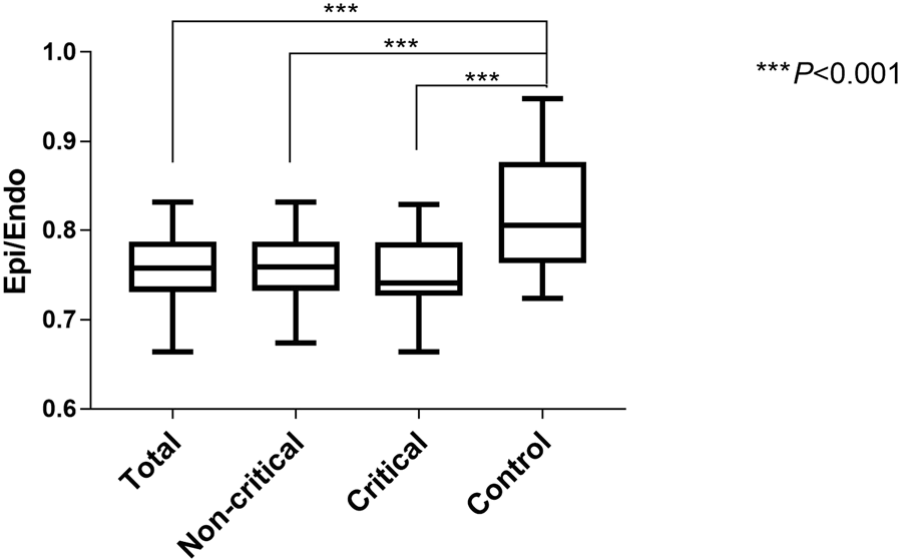
The reduction in longitudinal strain was more prominent in the subepicardial layer than in the subendocardial layer of the myocardium (***P  < 0.001 vs. control). GLSs global longitudinal strains, Endo subendocardium, Epi subepicardium, Epi/Endo the ratio of longitudinal strains of the subepicardial layer to longitudinal strains of the subendocardial layer.

**Fig. 4:**
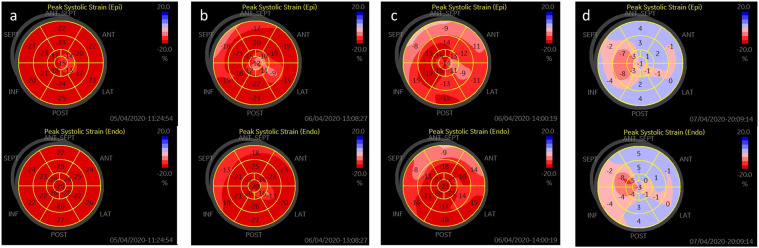
Representative images of GLS presented as “bull’s-eye”. (**a**) The subepicardium and the subendocardium images of a normal subject, (**b**) the subepicardium and the subendocardium images of a noncritical COVID-19 patient, (**c**) the subepicardium and the subendocardium images of a critical COVID-19 patient, (**d**) the subepicardium and the subendocardium images of a critical COVID-19 patient who died. The top line shows images of the subepicardium, and the bottom line shows images of the subendocardium. Alterations in layer-specific longitudinal strains were preferentially seen in the subepicardial layer of the myocardium. Endo subendocardium, Epi subepicardium.

Consistent with the reduction in GLS, both subendocardial (Endo) and subepicardial (Epi) strains were reduced in patients with COVID-19 in comparison with normal subjects. Again, the Endo and Epi strains were much lower in the critical patients than in the noncritical patients (Endo, −(16.8 ± 4.6)% vs. −(20.8 ± 3.5)%, respectively, *P* < 0.001; Epi, −(12.6 ± 3.6)% vs. −(15.8 ± 2.8)%, respectively, *P* < 0.001) (Table 2 and Fig. 1). The features of LSLS alterations were characterized by the Epi/Endo ratio, although the strains were lower in the subepicardium than in the subendocardium in normal subjects, and this difference was augmented in the COVID-19 patients. As a result, the ratio of Epi/Endo was significantly lower in the COVID-19 patients than in the normal controls (0.75 ± 0.04 vs. 0.82 ± 0.06, respectively, *P* < 0.001, Fig. 3), indicating preferential damage in the subepicardial layer of the myocardium in patients with COVID-19. Intriguingly, the ratio of Epi/Endo was only modestly lower in the critically ill patients than in the noncritical patients (0.74 ± 0.06 vs. 0.76 ± 0.04, respectively, *P* = 0.076, Table 2). These features were consistent with the presence of myocarditis [11, 18, 19]. Representative bull’s eye images of LSLS in a normal subject, a noncritical COVID-19 patient, a critical COVID-19 patient, and a COVID-19 patient who died are displayed in Fig. 4.

### Association of GLS changes with possible predictors

We performed linear regression analysis between GLS and different parameters, including troponin I, NT-proBNP, serum inflammatory markers (ESR and hsCRP), cytokines (IL-6, IL-10 and TNF-α), SpO_2_, and medical treatments (antiviral therapy, intravenous and oral use of corticosteroid, and dopamine/norepinephrine). Among the variables, age, troponin I, NT-proBNP, SpO_2_, hsCRP, IL-6, and TNF-α were all significantly associated with GLS changes (Table 3). Regarding the medical treatments, only intravenous use of corticosteroids was associated with GLS changes (Table 3). After adjusting for age and sex, cTnI, NT-proBNP, SpO_2_, hsCRP, IL-6, TNF-α, and intravenous use of corticosteroids remained correlated with GLS (RRs were −0.58, −0.37, 0.42, −0.20, −0.21, −0.16, and −0.59, respectively; and *P* values were <0.0001, <0.0001, <0.0001, 0.006, 0.003, 0.022, and 0.027, respectively).

**Table 3:** Univariate and multivariate analysis to test the association between GLS and different clinical parameters and medical treatments.

Variables	Univariate		Multivariable^a^	
	Relative risk (95% CI)	*P* value	Relative risk (95% CI)	*P* value
Age	0.18	0.007	–	–
Male	−0.10	0.142	–	–
cTnI	−0.56	<0.0001	−0.58	<0.0001
NT-proBNP	−0.37	<0.0001	−0.37	<0.0001
SpO_2_	0.43	<0.0001	0.42	<0.0001
hsCRP	−0.23	0.0002	−0.20	0.006
ESR	−0.15	0.094	−0.15	0.116
IL-6	−0.23	0.001	−0.21	0.003
IL-10	−0.13	0.079	−0.12	0.096
TNF-α	−0.19	0.006	−0.16	0.022
Antiviral treatment	−0.71	0.723	−0.73	0.344
Intravenous corticosteroid	−0.62	0.093	−0.59	0.027
Oral corticosteroid	−0.47	0.521	−0.48	0.346
Dopamine/norepinephrine	−0.16	>0.999	−63.3	1.000

^a^Correction for age and sex.

### Clinical outcomes

As of April 2, 2020, two patients (0.9%) from the study cohort had died. The present study started on March 15, 2020, which was after the peak point of the COVID-19 outbreak in Wuhan, which may explain the low mortality in the study. The median length of hospitalization was 28 (IQR: 16, 43) days; the median hospital stay was longer for critical patients than for the noncritical patients (27 (IQR: 14, 43) vs. 40 (IQR: 28, 45) days, respectively, *P* < 0.001). In the entire study cohort, 181 (83%) patients had a reduction in GLS at the threshold of <−21.0%, and 48 (22%) had LV EF < 50% (Table 1).

### Reproducibility

The ICC for strain measurement was 0.91 (95% CI: 0.85–0.93) for interobserver agreement and 0.94 (95% CI: 0.90–0.97) for intraobserver agreement, indicating good intraobserver and interobserver correlations.

## Discussion

To our knowledge, this is the first comprehensive analysis of cardiac function using 2-D STE in a large COVID-19 cohort. We found that almost all critically ill COVID-19 patients (98%) and most noncritical patients (78.3%) had detectable abnormalities of cardiac function using deformation analysis by 2-D STE. These findings demonstrated for the first time the presence of widespread cardiac dysfunction that might contribute to the prognosis of COVID-19 patients. Our results suggest that myocardial deformation indices are sensitive indicators of cardiac injury and seemingly superior to biomarkers cTnI and NT-proBNP in COVID-19 patients. We further found that a reduction in strain was predominantly detected in the subepicardium rather than the subendocardium, which was consistent with known features of myocardial damage from myocarditis. Moreover, the GLS parameters were significantly associated with the serum levels of inflammatory cytokines and SpO_2_, underscoring the potential mechanisms involved in the pathogenesis of COVID-19-induced cardiac injury and dysfunction.

Hypersensitive cTnI was found to be elevated in 10.8% of the COVID-19 cohort, a level comparable to that observed in a previous report [1]. Elevated NT-proBNP (900-pg/mL cutoff value) levels were observed in 15.3% of COVID-19 patients, slightly lower than the 22.2% reported in a previous study from Wuhan [4]. In addition, 22% of patients had a reduced LV EF (<50%), a commonly used indicator of LV function. In contrast, 83% of the COVID-19 patients had reduced GLS, which was more common than the prevalence of reduced LV EF or elevated cTnI and NT-proBNP. These data indicate that most hospitalized COVID-19 patients developed subclinical LV dysfunction, despite preserved EF and normal levels of cTnI and/or NT-proBNP. Our findings offer the first clinical evidence that cardiac abnormalities are a common finding in COVID-19, and their prevalence is much higher than that previously reported. New imaging tools, such as myocardial strain analysis by 2-D STE, provide a valuable approach to detect the full spectrum of cardiac abnormalities and may be considered as part of the diagnostic evaluation of patients with COVID-19, particularly critically ill patients.

Patients in this study were mostly in the mid- or late-phase of their disease course; therefore, symptoms such as fever and cough had resolved. The most common symptom among the cohort was shortness of breath, which could have been caused by either respiratory distress or cardiac dysfunction or both. However, the typical symptoms of heart failure, including orthopnea, paroxysmal nocturnal dyspnea, and ankle swelling, were largely absent in the study subjects. Likewise, in 94.5% of patients with cTnI elevation, the absolute cTnI level was lower than 100 pg/mL. The asymptomatic presentation of cardiac dysfunction and the low-grade elevation of cTnI were in line with the subclinical state of the cardiac dysfunction associated with COVID-19.

GLS reduction was more common in critically ill patients (98%) than in noncritical patients (78.3%). Likewise, the absolute levels of GLS were significantly lower in the critical cases than in the noncritical cases. This correlation suggests that cardiac dysfunction may have a significant contribution to disease progression and adverse outcomes in patients with COVID-19.

Histologic evidence of inflammatory cell infiltrates by EMB is the gold standard criterion for diagnosing myocarditis [20]. Likewise, CMR is an alternative noninvasive imaging tool [21]. However, EMB and CMR have not been routinely available during the COVID-19 pandemic, raising a major challenge for proper diagnosis of the state of cardiac health in patients with COVID-19. Myocardial deformation analysis by 2-D STE, particularly layer-specific quantification, provides a novel tool for detecting cardiac dysfunction in COVID-19 patients. Indices of longitudinal strains are known to strongly correlate with the levels of lymphocytic infiltrates in EMB samples [12] and with the amount of edema detected by CMR [13]. Moreover, the diagnostic performance of LSLSs in acute myocarditis has been validated by showing preferential alteration of subepicardial deformation that was consistent with tissue characteristics established by CMR [11]. In the present study, the reduction in longitudinal strains in the subepicardium was more severe than that in the subendocardium, suggesting that cardiac dysfunction was located predominantly in the subepicardial layer of the myocardium, an important feature consistent with the manifestation of myocarditis. Indeed, 18 patients from the present study cohort had CMR imaging after their discharge (data not shown), of whom 8 showed evidence of myocardial edema. Therefore, the findings of the present study of a large cohort provided additional evidence, based on echo imaging, to support the potential involvement of myocarditis in COVID-19.

Myocardial inflammation involves pericardial remodeling, and vice versa. In this study, we noted that 11.5% of COVID-19 patients developed pericardial effusion; however, most cases were mild, and only a few had moderate effusion. Large effusion was not detected, and no difference in the incidence of pericarditis was observed in the critical vs. the noncritical patients.

Whether SARS-CoV-2 can directly cause primary myocardial injury through myocardial inflammation is still controversial, though it has been widely speculated based on previous knowledge of other viral-induced cardiac injuries [22]. To date, there have been only two case reports indicating the presence of myocarditis by showing the presence of myocardial edema using CMR imaging [23, 24] and one case report detected the presence of SARS-CoV-2 in EMB sample tissue [25]. However, neither inflammatory infiltrates nor substantial myocardial damage was found in a patient who died from severe COVID-19 infection [26]. There was also no direct evidence of myocarditis in the COVID-19 cohorts at this moment.

In addition to myocardial inflammation, systemic inflammation and hypoxemia may also contribute to cardiac injury under different pathological conditions [27–31]. The systemic inflammatory response has been found to be associated with early cardiac dysfunction in patients with traumatic brain injury and in perioperative myocardial injury in patients undergoing noncardiac surgery [27, 29]. Inflammatory cytokines, such as IL-6, IL-10, and TNF-α, have been associated with cardiovascular dysfunction in patients with cardiac arrest [28]. Hypoxemia may also lead to cardiac injury via inflammation, metabolic acidosis, and mitochondrial abnormalities, as suggested by previous studies [30, 31]. However, their roles in cardiac injury and dysfunction in COVID-19 are not known. In the present study, the levels of serum inflammatory cytokines, such as IL-6, IL-10, and TNF-α, as well as inflammation markers, such as ESR and CRP, were all significantly elevated, especially in the critically ill patients. Hypoxemia was also particularly common in the critically ill patients. Therefore, we suspected that systemic inflammation and hypoxemia may cause secondary myocardial injury in COVID-19. By multivariable regression analysis, we found that systemic inflammatory cytokines, particularly IL-6 and SpO_2_, were closely associated with a reduction in GLS in COVID-19 patients. Therefore, we speculated that systemic inflammation and hypoxemia may contribute to cardiac injury in patients with COVID-19. Regarding the effects of medical treatment on myocardial injury, only the intravenous use of corticosteroids was associated with improvement in GLS, indicating the potential beneficial effect of systemic corticosteroid treatment. However, further evidence is needed to assess the value of corticosteroids in COVID-19.

### Limitations

There are some notable limitations in the present study. First, patients with underlying cardiovascular diseases were excluded, and important comorbidities, such as hypertension and diabetes, were not investigated. In addition, the normal cohort in this study consisted of only 23 subjects, which is modest and may complicate the interpretation of the findings on the effects of COVID-19 on cardiac function. The TTE scans in the study were performed at the time when most of the enrolled patients had passed the critical period of COVID-19 disease progression; most patients were generally toward the end of their hospitalization period. Therefore, the echocardiographic features obtained in the study may be subject to survival bias and may not be adequate to predict the outcome. Meanwhile, critically ill patients who died earlier than March 15 were not included in this study, which explained the low mortality in the present study and may affect the outcome analysis. Moreover, EMB data were absent in the present study. Therefore, although the study raised the possibility that COVID-19 was associated with highly prevalent cardiac abnormalities and could potentially cause myocarditis, direct evidence remains lacking. Fourth, different treatments may be significant confounding factors of the disease outcome and may have further complicated the statistical analysis and the conclusions of the study. Fifth, repeated echo and with serum markers were not performed at the end of hospitalization to determine the changes in cardiac injury. Last, whether COVID-19 could cause chronic cardiomyopathy was not addressed in this study, and long-term follow-up is needed.

## Conclusions

This study provides imaging evidence of the high prevalence of myocardial dysfunction in a large population of patients with COVID-19. Myocardial deformation analysis by 2-D STE detected a broad range of cardiac abnormalities in COVID-19 patients. The preferential alteration of strains in the subepicardium supports possible myocarditis as the underpinning cardiac pathology in COVID-19. Likewise, inflammatory storms and hypoxemia may be important mechanisms leading to cardiac injury in COVID-19 patients.
